# Harnessing liposomal delivery to boost polyphenols’ potential in chronic wound therapy

**DOI:** 10.3389/fddev.2026.1839747

**Published:** 2026-08-21

**Authors:** Lisa Myrseth Hemmingsen, Marte Kristensen, Cecilie Thanh Loan Dang, Mari Salamonsen, Elizabeth G. Aarag Fredheim, Nataša Škalko-Basnet

**Affiliations:** 1 Department of Pharmacy, Drug Transport and Delivery Research Group, University of Tromsø The Arctic University of Norway, Tromsø, Norway; 2 Department of Pharmacy, Natural Products and Medicinal Chemistry Research Group, University of Tromsø The Arctic University of Norway, Tromsø, Norway; 3 Department of Pharmacy, Microbial Pharmacology and Population Biology, University of Tromsø The Arctic University of Norway, Tromsø, Norway

**Keywords:** antimicrobial therapy, biocompatibility, chronic wounds, liposomes, polyphenols

## Abstract

**Introduction:**

Chronic wounds pose a significant clinical challenge due to impaired healing, persistent inflammation, and susceptibility to infection, often by antimicrobial-resistant bacteria. Therefore, active compounds should ideally act both as antimicrobials with limited potential for resistance development and offer other beneficial wound-healing properties. Polyphenols, such as chlorogenic acid (CGA) and quercetin (QCT), exhibit those properties, but are limited by poor stability or low bioavailability.

**Methods:**

In this study, liposomal formulations of CGA (CGA-LP) and QCT (QCT-LP) were tailored to overcome delivery barriers to wound sites. Liposomes were characterized by size, size distribution, zeta potential, entrapment efficiency, and stability.

**Results and Discussion:**

*In vitro* release studies for CGA-LP and QCT-LP demonstrated sustained payload release of ∼30% and ∼50%, respectively, over 24 h. Antioxidant activity of both compounds was assessed using DPPH and ABTS·^+^ assays, confirming the radical scavenging potential of CGA comparable to vitamins C and E, while QCT showed high DPPH activity, but limited ABTS·^+^ activity. Biocompatibility studies on murine macrophages revealed no cytotoxicity for either formulation. Antibacterial activity was assessed via broth microdilution for both formulations and isothermal microcalorimetry for CGA-LPs. Only QCT-LP potent measurable inhibitory effects against *Staphylococcus aureus* in the broth microdilution assay; however, effects on metabolic activity were observed for CGA-LPs. These findings suggest that liposomal entrapment could protect polyphenols and prolong or tailor their release, potentially offering a promising strategy for chronic wound therapy and addressing challenges associated with oxidative stress and bacterial infections.

## Graphical Abstract

**Figure:**
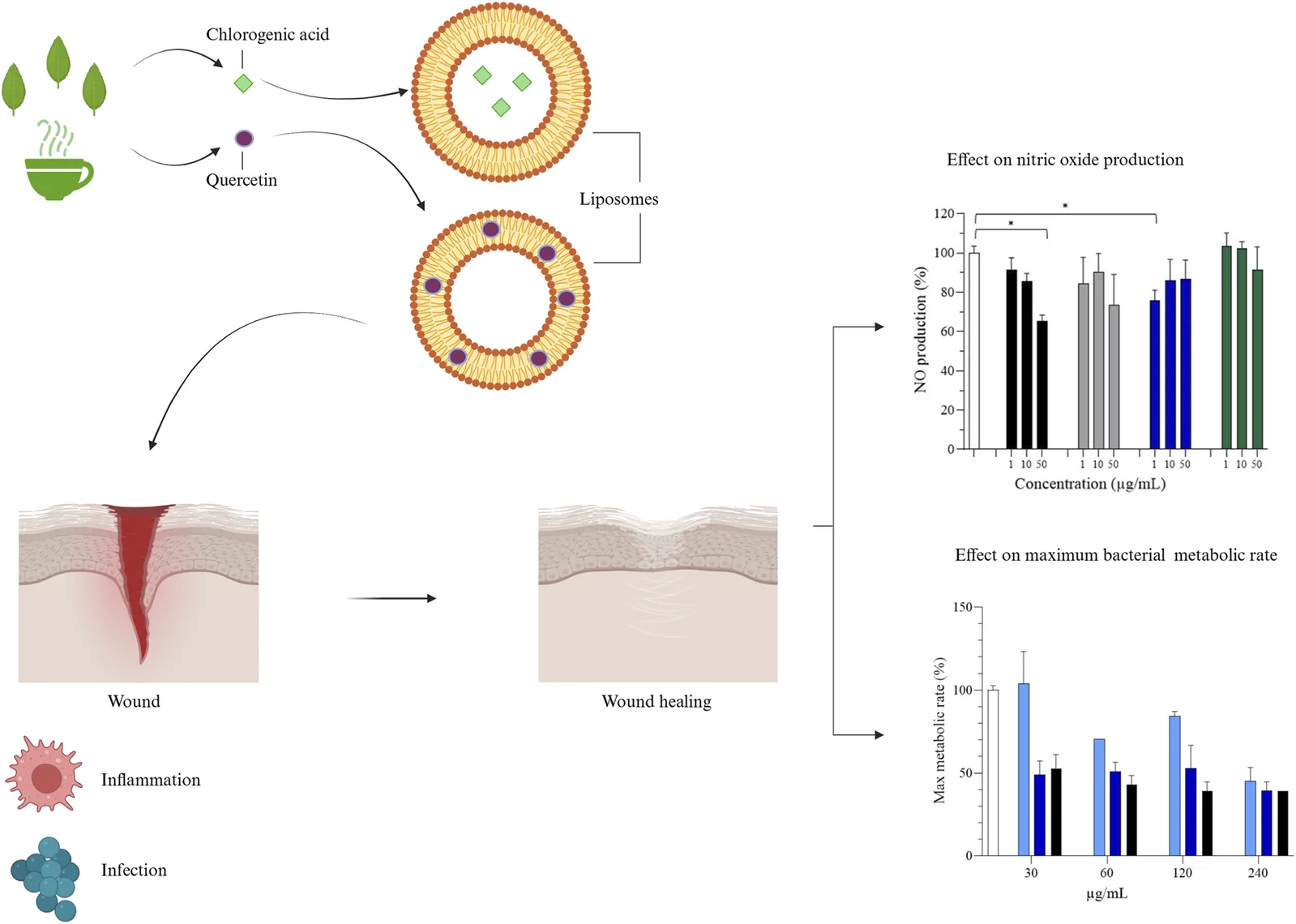

## 1 Introduction

Chronic wounds represent a major healthcare challenge worldwide, often described as a silent epidemic, as they significantly impair patient quality of life and place a substantial economic burden on healthcare systems (Kolluri et al., 2022; Sharma et al., 2024). Patients with chronic wounds experience severe pain, reduced mobility, emotional distress, and are at increased risk of infections due to breaches in the skin barrier and impaired healing processes (Falanga et al., 2022; Tomazoni and Nihei, 2025). The prevalence of chronic wounds is rising in parallel with aging populations and the increasing incidence of lifestyle-related conditions such as diabetes and obesity (Sen, 2023). Infected wounds are often colonized by multiple bacterial species, fungi, and viruses, and are challenging to treat due to the widespread emergence of antimicrobial resistance, which reduces the efficacy of conventional antibiotics and complicates clinical management (Falcone et al., 2021; Tang et al., 2023; Uberoi et al., 2024).

Historically, natural compounds have been used to treat skin infections and promote wound healing due to their antimicrobial and anti-inflammatory properties (Nazari et al., 2025). Polyphenols, including chlorogenic acid (CGA) and quercetin (QCT), have gained significant interest for their potential therapeutic roles in wound management. Notably, CGA exhibits antioxidative, anti-inflammatory, and antibacterial activities (Huang J. et al., 2023), whereas QCT is a flavonoid with broad pharmacological effects, including antioxidative, antibacterial, anti-inflammatory, anti-cancer, and cardioprotective properties (Shabir et al., 2022). Moreover, QCT has also been shown to modulate oxidative stress, inflammatory responses, and cellular signalling pathways, highlighting its potential as a therapeutic compound (Dandamudi et al., 2024). Despite their promising bioactivity, both compounds face limitations such as poor water solubility, low stability, or limited dermal bioavailability, which restrict their clinical use (Hu et al., 2024).

Liposomal entrapment offers a compelling strategy to overcome these limitations. Liposomes are self-assembled spherical vesicles composed of lipid bilayers surrounding an aqueous core, capable of carrying both hydrophilic and hydrophobic compounds (Luo et al., 2025). Their amphiphilic nature and similarity to biological membranes could provide high biocompatibility, low cytotoxicity, and non-irritant properties, making them suitable for application to damaged or inflamed skin, including chronic wounds (Nsairat et al., 2022; Dragicevic and Maibach, 2024). Liposomal delivery systems could improve the stability and solubility of polyphenols, protect them from premature degradation, and allow for sustained release, which might enhance antimicrobial efficacy and therapeutic outcomes (Aytekin et al., 2020; Giordani et al., 2020; Trivedi and Puranik, 2023). Furthermore, carriers like liposomes could support targeted delivery to the wound site, potentially maintaining effective concentrations of active compounds over extended periods and reducing the frequency of administration, which might improve patient compliance and reduce treatment costs (Raina et al., 2023). Compared to conventional topical wound therapies, liposomal formulations could additionally provide improved localization and retention of compounds at the wound site while minimizing premature degradation and uncontrolled diffusion. These properties might be particularly advantageous in chronic wound management, where prolonged inflammation, oxidative stress, microbial burden, and frequent dressing changes could limit the therapeutic effectiveness of standard treatments (Hurlow and Bowler, 2022).

In this study, we developed CGA-loaded liposomes (CGA-LP) and QCT-loaded liposomes (QCT-LP) designed to enhance the stability, biocompatibility, and antimicrobial activity of these compounds, thereby improving their therapeutic potential against typical wound pathogens such as *Staphylococcus aureus* (Wolcott et al., 2016). The primary focus of this study was the formulation development and initial antimicrobial evaluation of these liposomal systems as natural, multitargeting therapeutic candidates for chronic wound management.

## 2 Materials and methods

### 2.1 Materials

#### 2.1.1 Materials

Lipoid S100 (>94% phosphatidylcholine from soybean) was kindly provided by Lipoid GmbH (Ludwigshafen, Germany). 2,2′-Azino-bis(3-ethylbenzothiazoline-6-sulfonic acid) diammonium salt (ABTS·^+^), chlorogenic acid (≥95%), quercetin (≥95%, HPLC), α-tocopherol (≥95%, HPLC), L-ascorbic acid, propylene glycol, Cell Counting Kit-8 (CCK-8), penicillin-streptomycin, fetal bovine serum (FBS) suitable for cell culture, potassium peroxodisulfate, and 2,2-Diphenyl-1-picrylhydrazyl (DPPH), and RPMI-1640 medium with L-glutamine and sodium bicarbonate, sterile-filtered, suitable for cell culture were obtained from Sigma-Aldrich (St. Louis, USA). Acetonitrile (≥99.95%) and methanol (gradient grade, for HPLC) were purchased from VWR International (Fontenay-sous-Bois, France). Formic acid (98%–100%) was purchased from Merck KGaA (Darmstadt, Germany). Sodium Chloride (1.06404) was used for saline solutions and obtained from Merck KGaA (Darmstadt, Germany). Dimethyl sulfoxide (DMSO) cell culture reagent was obtained from MP Biomedicals (Fountain Parkway, USA). Mueller-Hinton II Broth (212322) was used for MHII broth and obtained from BD (Franklin Lakes, USA). Blood Agar Base No. 2 (CM0271) and Defibrinated Horse Blood (SR0050) were used for blood agar plates and obtained from Thermo Fisher (Hampshire, UK).

#### 2.1.2 Bacterial strain, cell lines, and culture conditions

*Staphylococcus aureus* MSSA 476 (ATCC® BAA-1721™) was obtained from LGC Standards AB (Borås, Sweden) and cultivated on blood agar plates under standard culturing conditions at 37 °C over night in preparation of testing. Murine macrophage cell line RAW 264.7 was obtained from ATCC (Manassas, USA) and cultured in RPMI-1640 supplemented with 10% (v/v) FBS and 1% (v/v) penicillin-streptomycin at 37 °C in a 5% CO_2_ atmosphere.

### 2.2 Liposome preparation

#### 2.2.1 Liposome production

Empty and loaded liposomes were prepared by the thin-film hydration method (Braido et al., 2025). Briefly, Lipoid S100 (200 mg) was transferred to a 50 mL round-bottom flask and dissolved in methanol with hand stirring until a clear solution was obtained. For QCT-loaded liposomes (QCT-LP), 10 mg QCT was added to the round-bottom flask prior to dissolution, whereas for CGA-loaded liposomes (CGA-LP), the lipid film was rehydrated with 10 mL distilled water containing 20 mg CGA. The solvent was evaporated using a Büchi Rotavapor, with the water bath set at 45 °C and rotation speed at 60 rpm, while the pressure was gradually reduced to form a thin lipid film. To remove residual solvent, the flask was rotated at 60 mBar under pressure for at least 1 h. The dry lipid film was then rehydrated with 10 mL of distilled water (for empty and QCT-LP) or CGA solution in distilled water (for CGA-LP) and hand-shaken to fully dislodge the film. The resulting liposome suspensions were stored at 4 °C–8 °C overnight prior to further experiments.

#### 2.2.2 Size reduction

The size of liposomes was reduced using either membrane extrusion or probe sonication, depending on the physicochemical properties of the encapsulated compound and the suitability of the processing method for the resulting formulation (Hemmingsen et al., 2022). Consequently, CGA, a relatively hydrophilic compound, was processed by membrane extrusion to achieve size reduction while minimising potential payload loss. In contrast, QCT, which is poorly water-soluble and expected to partition predominantly within the lipid bilayer, was processed by probe sonication, as this method proved more suitable during formulation development. The CGA-LP and their corresponding empty liposomes (Empty-LP_Ext._) were manually extruded through polycarbonate membrane filters. Liposome suspensions were sequentially passed through 0.8 µm (three times), 0.4 µm (five times), and 0.2 µm (three times) membranes to achieve the desired size. The QCT-LP and their corresponding empty liposomes (Empty-LP_Probe_) were subjected to size reduction via probe sonication (SONICS high intensity ultrasonic processor, 500-watt model, 13 mm probe diameter, Sonics & Materials Inc., Newtown, CT, USA). During sonication, dispersions were placed in an ice bath to prevent extensive heating, and the probe was positioned centrally just below the dispersion surface. The sonicator was set to 40% amplitude with 10-s pulses. The QCT-LP were sonicated for 29 cycles, and Empty-LP_Probe_ for 31 cycles, with 20-s pauses between cycles to avoid overheating. All liposome suspensions were stored at 4 °C–8 °C overnight prior to further experiments.

### 2.3 Characterisation of liposomes

#### 2.3.1 Liposome size and zeta potential determination

The size, polydispersity index (PI), and zeta potential of all liposome batches were determined using dynamic light scattering. The size of Empty-LP_Ext_ and CGA-LP was measured on a Zetasizer Nano Zen 2600 (Malvern, Worcestershire, UK) at 25 °C after dilution 1:100 (v/v) with filtered (0.2 µm) water (Braido et al., 2025). The QCT-LP and Empty-LP_Probe_ were analysed using a NICOMP Submicron Particle Sizer Model 370 (NICOMP Particle Sizing system, Santa Barbara, CA, USA), as probe-sonicated liposomes exhibited broader and potentially multimodal size distributions, for which the NICOMP system was considered more suitable, and their dilutions were adjusted to achieve an intensity of 250–350 kHz in prerinsed disposable drop cells prior to measurement (Hupfeld et al., 2006). Size measurements were performed in three cycles of 30 min each at 23 °C ± 1 °C with a scattering angle of 90°. Zeta potential measurements for all liposome batches were performed on the Zetasizer Nano Zen 2600 using folded capillary cells. Prior to measurement, each sample was diluted 1:20 (v/v) with filtered (0.2 µm) water and measured in three parallel cycles at 25 °C. All measurements were performed in triplicates.

#### 2.3.2 Determination of entrapment efficiency

The entrapment efficiencies (EE%) were determined by quantifying both liposomally-entrapped and unentrapped compounds. For CGA-LP, free CGA was separated from the entrapped fraction using the dialysis method. Briefly, 1 mL of liposome suspension was enclosed in a dialysis membrane with a molecular weight cutoff of 12–14 kDa (Spectra/Por®4, Spectrum®, VWR International, Fontenay-sous-Bois, France) and placed in 100 mL of distilled water under magnetic stirring for four hours at room temperature (23 °C–25 °C). Absorbance measurements were performed using a SPARK® multimode microplate reader (Tecan Trading AG, Männedorf, Switzerland) at 330 nm. For QCT-LP, free QCT was separated from the entrapped fraction using centrifugation due to solubility limitations in water (Hemmingsen et al., 2021). Samples were centrifuged at 3,000 *g* for 15 min at room temperature on the Biofuge Stratos centrifuge (Heraeus Instruments GmbH, Hanau, Germany). Samples collected before and after centrifugation were further diluted in methanol, and the entrapped QCT was quantified using the SPARK® multimode microplate reader at 370 nm. The EE % of both compounds was calculated using Equation 1:

$$EE\%=Y_{1}/Y_{0}\times 100\;\%$$ (1)

where Y_1_ represents the amount of the compound after separation and Y_0_ represents the amount of the compound before separation. The EE% was calculated for three replicates.

#### 2.3.3 Liposome stability

The storage stability of Empty-LP_Ext._, Empty-LP_Probe_, CGA-LP, and QCT-LP was evaluated after 4 weeks at 4 °C–8 °C with respect to their physicochemical properties, including size, size distribution, and zeta potential as described in Section 2.3.1.

### 2.4 *In vitro* release studies

#### 2.4.1 Franz diffusion release studies

The *in vitro* release of CGA from CGA-LP and QCT from QCT-LP was assessed using a Franz diffusion cell system equipped with a heating circulator to maintain 32 °C, simulating skin surface temperature as previously described, with minor modifications (Braido et al., 2025). For CGA-LP, the acceptor chambers (5 mL, diffusion area 0.64 cm^2^) were filled with distilled water, whereas for QCT-LP, the acceptor chambers (12 mL, diffusion area 1.77 cm^2^) were filled with methanol due to the low water solubility of QCT. Donor chambers were loaded with 600 µL of liposome formulations, and control solutions containing CGA or QCT at comparable concentrations were included. Stirring was applied in the acceptor chambers, and the sampling port and donor chamber were covered to prevent evaporation. Samples (500 µL) were withdrawn from the acceptor chamber at predetermined time points. After each sampling, the withdrawn volume was immediately replaced with fresh acceptor medium to maintain sink conditions. The amount of CGA and QCT in the samples was quantified using ultra-performance liquid chromatography coupled with ultraviolet detection (UPLC-UV) as described in Section 2.4.2.

#### 2.4.2 Quantification

The concentrations of CGA and QCT released from liposomes were determined using UPLC-UV. A sample volume of 5 µL was injected onto an Acquity UPLC® BEH C18 column (100 mm × 2.1 mm, 1.7 µm; Waters Corporation, Milford, MA, USA) using an Acquity UPLC H-class system equipped with a PDA detector (Waters Corp.). Mobile phase A consisted of Milli-Q water with 0.1% (v/v) formic acid, and mobile phase B consisted of acetonitrile with 0.1% (v/v) formic acid. Gradient elution was performed, increasing from 10% to 40% B over 5 min. The flow rate was 0.6 mL/min, the column temperature was maintained at 60 °C, and detection was performed at 325 nm and 370 nm for CGA and QCT, respectively.

### 2.5 Radical scavenging assessments

#### 2.5.1 DPPH radical scavenging

The antioxidant activity of CGA and QCT was evaluated using the lipophilic DPPH radical scavenging assay, following the method of Jøraholmen *et al.* (Jøraholmen et al., 2019). A 60 µM DPPH solution was mixed in equal volumes with CGA or QCT solutions at final concentrations of 5, 10, 25, 50, and 75 µM. The mixtures were kept in the dark for 30 min at room temperature. Subsequently, aliquots from each sample were transferred to a UV plate and measured spectrophotometrically at 519 nm using the SPARK® multimode microplate reader (Tecan Trading AG, Männedorf, Switzerland). The antioxidative activity of CGA and QCT was compared to that of known antioxidants, vitamins C and E, under the same conditions and corresponding concentrations. The percentage of radical scavenging activity was calculated using the Equation 2:

$$Scavenging\;activity\;\left(\%\right)=\left(S_{control}-S_{Sample}\right)/S_{control}\times 100\;\%$$ (2)

where S_control_ is the absorbance of the radical solution without sample and S_sample_ is the absorbance in the presence of the test compound.

#### 2.5.2 ABTS·^+^ radical scavenging

The antioxidant activity of CGA and QCT was also evaluated using the hydrophilic-compatible ABTS·^+^ radical scavenging assay, following a modified method of (Jøraholmen et al., 2019). The ABTS·^+^ radical solution was prepared by mixing 3 mL of ABTS·^+^ stock solution (7.4 mM) with potassium peroxodisulfate (2.6 mM) and storing the mixture overnight at room temperature in the dark. The radical solution was then diluted to 100 mL with ethanol and mixed in equal volumes with CGA or QCT solutions at final concentrations of 5, 10, 25, 50, and 75 µM. The mixtures were kept in the dark for 30 min at room temperature, after which aliquots were transferred to a UV plate and measured spectrophotometrically at 731 nm using the SPARK® multimode microplate reader (Tecan Trading AG, Männedorf, Switzerland). The antioxidative activity of CGA and QCT was compared to that of known antioxidants, vitamins C and E, under the same conditions and corresponding concentrations. A decrease in absorbance was used to determine radical scavenging activity. The percentage of radical scavenging activity was calculated using Equation 2 in Section 2.5.1.

### 2.6 Toxicity evaluation

The cytotoxicity of liposomal formulations was assessed in murine macrophages RAW 264.7 using the Cell Counting Kit-8 (CCK-8), following a previously described protocol (Hemmingsen et al., 2022). Briefly, 1 × 10^5^ cells/mL was seeded into 96-well plates and incubated for 24 h. Subsequently, 10 µL of medium (control) or diluted liposome suspensions corresponding to final lipid concentrations of 1, 10, or 50 μg/mL was added to the wells, corresponding to approximately 0.023, 0.23, and 1.15 μg/mL CGA, and 0.049, 0.49, and 2.45 μg/mL QCT, followed by an additional 24-h incubation. After incubation, 10 µL of CCK-8 reagent was added to each well, and the plates were incubated for 4 h prior to measurement of absorbance at 450 nm with a reference wavelength of 650 nm using the SPARK® multimode microplate reader (Tecan Trading AG, Männedorf, Switzerland). All formulations were tested in triplicate, and cytotoxicity was expressed as a percentage surviving macrophages relative to the control (untreated cells).

### 2.7 Anti-inflammatory assessment

The anti-inflammatory activity of the formulations was evaluated by measuring lipopolysaccharide (LPS)-induced nitric oxide (NO) production in murine macrophage RAW 264.7 cells, following a previously reported method (Hemmingsen et al., 2021). Briefly, cells were seeded into 24-well plates, followed by incubation for 24 h. After incubation, the culture medium was replaced with fresh medium containing LPS (1 μg/mL), and the cells were subsequently treated with liposomal suspensions corresponding to final lipid concentrations of 1, 10, or 50 μg/mL, corresponding to approximately 0.023, 0.23, and 1.15 μg/mL CGA, and 0.049, 0.49, and 2.45 μg/mL QCT. Cells treated with LPS-containing medium alone and complete medium without LPS served as positive and negative controls, respectively. Following an additional 24-h incubation under the same conditions, NO production was quantified using the Griess reagent (2.5% phosphoric acid containing 1% sulphanilamide and 0.1% N-(1-naphthyl)ethylenediamine). Absorbance was measured at 540 nm using the SPARK® multimode microplate reader (Tecan Trading AG, Männedorf, Switzerland). All experiments were performed in triplicate, and NO production was expressed relative to the LPS-treated control.

### 2.8 Antimicrobial evaluation

#### 2.8.1 Broth microdilution

The antibacterial activity of free polyphenols, Empty-LP_Ext_, Empty-LP_Probe_, CGA-LP, and QCT-LP against *S. aureus* MSSA 476 was evaluated using the broth microdilution method as described by Balouiri *et al.* (Balouiri et al., 2016). A twofold dilution series (10 mg/mL theoretical lipid concentration) in Mueller Hinton Broth (MHB) was prepared in 96-well plates and inoculated with equal volumes of bacterial suspension to an end concentration of 5 × 10^5^ CFU/mL. The plates were covered with parafilm to prevent evaporation and incubated at 37 °C with shaking (100 rpm) for 24 h. Bacterial survival was assessed by visual examination as described by CLSI (Clinical Laboratory Standards Institute, 2012), and absorbance was measured at 600 nm using the SPARK® multimode microplate reader (Tecan Trading AG, Männedorf, Switzerland).

#### 2.8.2 Isothermal microcalorimetry

The antibacterial activity of Empty-LP_Ext._, CGA-LP, and free CGA against *S. aureus* MSSA 476 was further investigated using isothermal microcalorimetry (IMC) as described by Tellapragada *et al.* (Tellapragada et al., 2020), with minor modifications. Four different concentrations were prepared by twofold dilutions in MHB, starting from 240 μg/mL CGA for CGA-LP and free CGA, and the corresponding theoretical lipid concentration of 10 mg/mL for the Empty-LP_Ext_. Further, the samples were inoculated with a suspension of MSSA 476 to a final concentration of 5 × 10^5^ CFU/mL and transferred to sterile plastic calWells™ without surface treatment and placed into titanium vials of the calPlate™ according to the manufacturer’s description. Wells containing only bacterial inoculum in MHB served as growth controls. Thermodynamic reference calWells™ containing MHB were included on each plate. The calPlate™ was sealed and incubated in the CalScreener™ (Symcel, Stockholm, Sweden) at 37 °C for 24 h to monitor bacterial growth through heat flow measurements. Activity was recorded as total metabolic rate (µW) and maximum metabolic rate (µW).

### 2.9 Statistical analysis

Results are expressed as mean ± Standard Deviation (SD). Student’s t-tests or one-way ANOVA with Tukey *post hoc* test were performed to evaluate significance (*p* < 0.05). All statistical analyses were performed in GraphPad Prism version 10.6.1 for Windows (GraphPad Software LLC, San Diego, CA, USA).

## 3 Results and discussion

### 3.1 Liposome characteristics

Liposomes were characterized by vesicle diameter, PI, and zeta potential (Table 1). All formulations were within the target range of 200–300 nm, which is favourable for dermal therapy (Mardhiah Adib et al., 2016). Although the link between vesicle size and skin penetration is not fully established and is affected by various characteristics (Akombaetwa et al., 2023; Guillot et al., 2023), vesicles in this range enhance retention within residual skin structures and wound tissue, as well as support penetration into bacterial biofilms (Meers et al., 2008; Li et al., 2025). The thin-film hydration method was selected because of its versatility, widespread use in liposome formulation, and suitability for incorporating lipophilic compounds into phospholipid bilayers (Kamini and Puri, 2026; Thabet et al., 2022). Although the method initially produces heterogeneous vesicle populations, subsequent extrusion or sonication enables control of vesicle size and size distribution for dermal delivery applications. Furthermore, using the same preparation method for all formulations enabled a more consistent comparison of the physicochemical properties and behaviour of the individual compounds within the liposomal systems. The CGA-LP and QCT-LP remained relatively stable in size over 4 weeks. However, in contrast, the two empty liposome formulations showed different behaviour. Namely, Empty-LP_Ext._ increased noticeably in mean diameter, consistent with aggregation or fusion during storage, whereas Empty-LP_Probe_ decreased in mean diameter, and significantly narrowed in distribution, suggesting a potential re-organisation or stabilization of the bilayers. Although not investigated directly, these differences partly reflect the different processing methods. In particular, probe sonication could influence lipid bilayer organisation and liposomal behaviour during storage, potentially contributing to the contrasting performance observed for the empty liposome formulations. After 4 weeks most formulations exhibited low PIs (≤0.24), however, QCT-LP retained the broader distribution. The improved stability observed for the CGA-LP and QCT-LP formulations compared to empty liposomes might also be related to the presence of polyphenols within the lipid bilayer. Polyphenolic compounds such as CGA and QCT are reported to interact with phospholipid headgroups and interfacial regions through hydrogen bonding and polar interactions, which could lead to increased membrane cohesion and reduced bilayer fluidity. Such interactions might in turn limit vesicle fusion or aggregation during storage, thereby contributing to the enhanced colloidal stability observed in the loaded formulations (Karonen, 2022; Wang et al., 2022; Xiang et al., 2025). Surface charge characteristics moved modestly toward more negative values for all liposomal formulations. The modest shift toward more negative surface charges could reflect slight lipid oxidation over time, which has been reported to alter the orientation of lipid polar head groups and affect how the liposome surface interacts with surrounding ions (Cauzzo et al., 2020). While empty liposomes remained near neutral, between −10 and 10 mV (Smith et al., 2017), reflecting the phosphatidylcholine composition of the liposomes, CGA-LP exhibited a more negative zeta potential. In addition to the factors discussed above, this shift might be related to the presence of CGA, which carries a negative charge at near-neutral pH and could therefore contribute to the surface charge through interactions with the liposomes.

**TABLE 1:** Physicochemical properties (size, polydispersity index, and zeta potential) of liposome formulations at baseline and after 4 weeks of storage.

Formulation	Week	Size (nm)	PI^a^	Zeta potential (mV)
Empty-LP_Ext_	0	212 ± 2	0.23 ± 0.01	−0.2 ± 0.7
	4	336 ± 35	0.24 ± 0.03	−3.0 ± 1.2
Empty-LP_Probe_	0	291 ± 63	0.47 ± 0.04	−0.1 ± 1.9
	4	233 ± 14	0.24 ± 0.03	−3.2 ± 0.1
CGA-LP	0	205 ± 34	0.16 ± 0.05	−8.6 ± 0.8
	4	200 ± 28	0.14 ± 0.05	−13.8 ± 1.7
QCT-LP	0	232 ± 38	0.36 ± 0.12	−8.3 ± 0.2
	4	239 ± 29	0.35 ± 0.05	−13.2 ± 3.3

Results are expressed as means with their respective SDs (*n* = 3).

^a^ Polydispersity index.

The EE% (Table 2) varied significantly between the compounds, as might be anticipated due to differences in their physicochemical properties, including hydrophobicity and chemical structure, which could influence incorporation into the liposomal bilayer. The CGA-LP showed a low EE%, which is commonly observed for small hydrophilic molecules entrapped in the aqueous core (Eloy et al., 2014). Li *et al.* reported a higher EE%, of about 50%, for CGA-LPs containing cholesterol prepared by ethanol injection, illustrating that lipid composition and preparation method might affect loading (Li et al., 2021). Although hydrophilic in nature, CGA is an unstable molecule. Liposomal encapsulation is therefore used to protect it from chemical degradation and to improve its stability and bioavailability by shielding it from environmental stressors and metabolic breakdown in biological systems. This approach has been shown to enhance both the stability and bioavailability of CGA compared with its free form (Li et al., 2024). Hong *et al.* reported an EE% of 17% when entrapping *Inula britannica* extract rich in CGA, which is comparable to our result (Hong et al., 2023).

**TABLE 2:** Entrapment efficiency of polyphenols in liposomal formulations.

Liposomal formulation	EE%^b^
CGA-LP	23.0 ± 3.1
QCT-LP	98.0 ± 12.7

Results are expressed as means with their respective SDs (*n* = 3).

^b^ Entrapment efficiency (%).

In contrast, QCT-LP exhibited high EE%, consistent with the lipophilic nature of QCT and its potential incorporation in the lipid bilayer. Similarly, high EE% were reported by Giordani *et al.* for QCT-LPs prepared by thin-film hydration and probe sonication (Giordani et al., 2020). Such lipophilic molecule requires formulation that would allow its solubilization to be able to exert biological activity.

### 3.2 *In vitro* release

**FIGURE 1:**
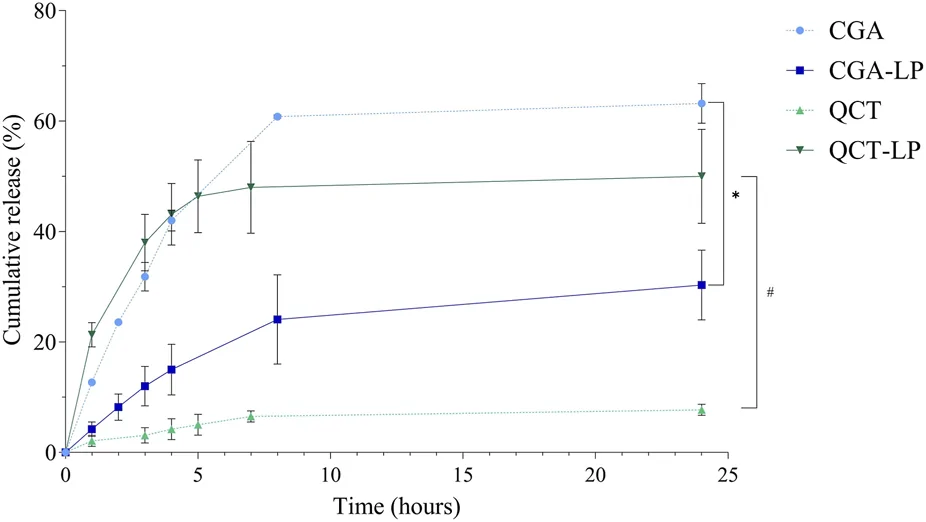
Cumulative *in vitro* release of CGA and QCT from solution or liposomes over 24 h at 32 °C, respectively. Results are expressed as means with their respective SD (*n* = 3). *p ≤ 0.05 between CGA and CGA-LP at 24 h ^#^p ≤ 0.05 between QCT and QCT-LP at 24 h.

Liposomes offer several advantages as drug delivery systems, including prolonged or sustained release of incorporated compounds or drugs. In this study, the *in vitro* release of CGA from CGA-LP was assessed and compared to permeation of free CGA dissolved in distilled water. Free CGA permeated rapidly, with ∼60% permeated within 24 h, whereas CGA-LP displayed a more prolonged release profile, with ∼30% released over the same period (Figure 1). The slower release from liposomes is attributable to diffusion of CGA through the lipid bilayer, which provides more consistent exposure, a sought property for chronic wound therapy, where prolonged therapeutic concentrations are needed (Wang et al., 2019; Ferreira et al., 2021). Furthermore, sustained release might also maintain concentrations at the infections site for an extended period, potentially enhancing or prolonging antimicrobial activity while minimizing adverse or off-target effects (Li et al., 2013; Kapoor et al., 2025).

Similarly, QCT-LP also exhibited prolonged release, with ∼50% released over 24 h, whereas free QCT in methanol showed lower permeation (∼13%). Methanol was required as the acceptor medium due to the low aqueous solubility of QCT. The initial faster release of QCT during the first five hours was followed by a more sustained profile, suggesting effective entrapment within the lipid bilayer. These findings align with studies on other polyphenols. Jøraholmen *et al.* reported prolonged release of resveratrol and epicatechin from liposomes, with ∼50–60% released after 8 h, whereas liposome-in-hydrogel formulations showed slower release due to the hydrogel matrix (Jøraholmen et al., 2019). The release profiles observed for CGA-LP and QCT-LP indicate that liposomal entrapment could modulate the release of hydrophilic and lipophilic compounds, respectively, potentially ensuring prolonged activity at the wound site. This might also reduce application frequency, improve patient compliance, and enhance therapeutic outcomes, consistent with previous findings on flavonoid-loaded liposomes in topical applications (Chen et al., 2014; Jøraholmen et al., 2019; Wang Z. et al., 2021). Although size of liposomes might influence release, the relatively similar size distributions observed for the loaded formulations suggest that the differences in release behaviour were more likely associated with compound-specific interactions with the lipid bilayer rather than liposome size.

### 3.3 Radical scavenging

**FIGURE 2:**
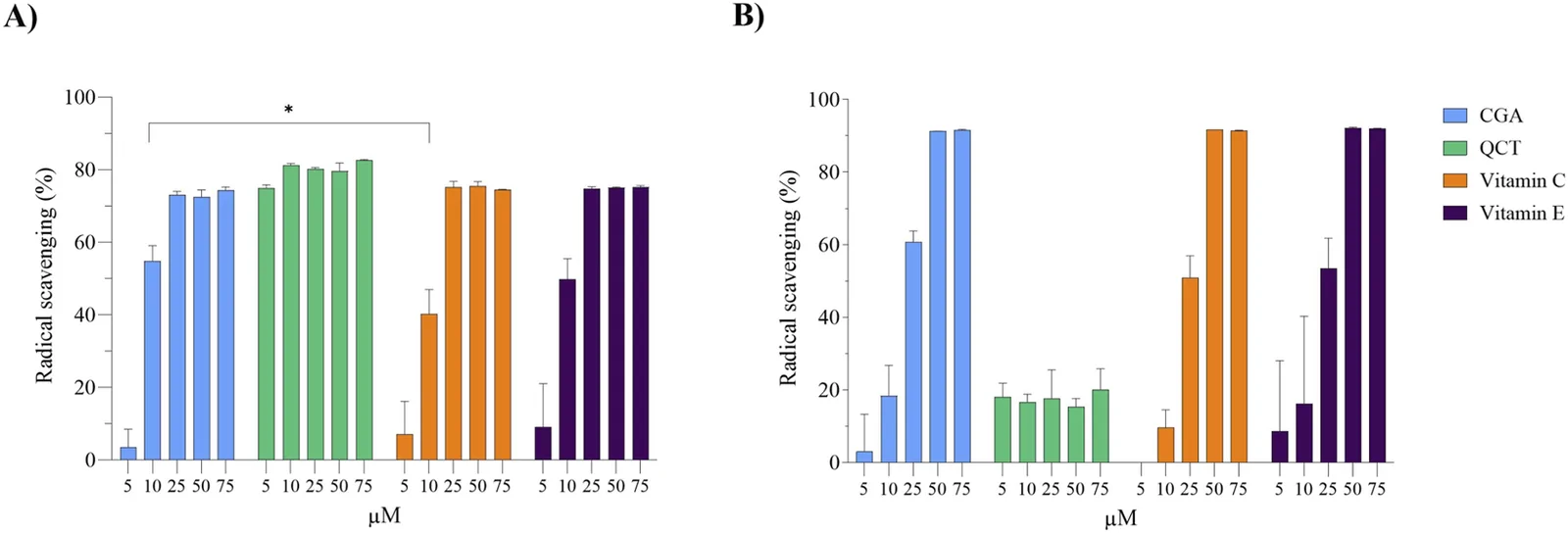
Antioxidative activities of CGA, QCT with vitamin C and E as controls. **(A)** DPPH radical scavenging assay and **(B)** ABTS·^+^ radical scavenging assay. The compounds were tested at final concentrations of 5–75 μM, with vitamins C and E prepared at equivalent concentrations to CGA and QCT. Data are presented as mean ± SD displayed as error bars (*n* = 3) *p ≤ 0.05.

Polyphenols are well-known for their antioxidative properties, which are relevant in wound healing due to their ability to neutralize reactive oxygen species (ROS) that could impair tissue repair (Comino-Sanz et al., 2021). The antioxidative activity of CGA and QCT was assessed in DPPH (Figure 2A) and ABTS·^+^ (Figure 2B) radical scavenging assays, with vitamins C and E serving as reference antioxidants. First, CGA exhibited concentration-dependent scavenging activity between 5 and 25 μM, reaching a plateau at higher concentrations (25–75 µM), and showed higher activity than vitamin C at 10 μM, consistent with earlier reports (Ohnishi et al., 1994; Izu et al., 2024). The ABTS·^+^ assays confirmed a similar trend, with all antioxidants demonstrating radical inhibition at higher concentrations, in line with findings of Choi *et al.* and Wang *et al.* (Choi et al., 2020; Wang S. et al., 2021).

On the other hand, QCT exhibited high DPPH scavenging activity across the tested concentration range, although a clear concentration-dependent relationship was not observed. This strong response might be partly attributed to the lipophilic nature of the DPPH radical, which could favour interactions with hydrophobic compounds such as QCT. At concentrations ≥5 μM, scavenging was comparable to literature values for flavonoids such as epicatechin (Jøraholmen et al., 2019) and QCT-liposomes (Giordani et al., 2020). However, only QCT reached ∼20% scavenging in the ABTS·^+^ assay regardless of the concentration. Given the well-established antioxidative activity of QCT in ABTS-based assays, this response is comparatively low. The observed difference might reflect the distinct physicochemical properties of the ABTS·^+^ assay, which is more compatible with hydrophilic antioxidants, whereas the DPPH assay is more selective toward lipophilic environments. In addition, interactions between QCT and formulation components might have affected the accessibility of the radical-scavenging groups under the assay conditions.

### 3.4 Evaluation of toxicity

**FIGURE 3:**
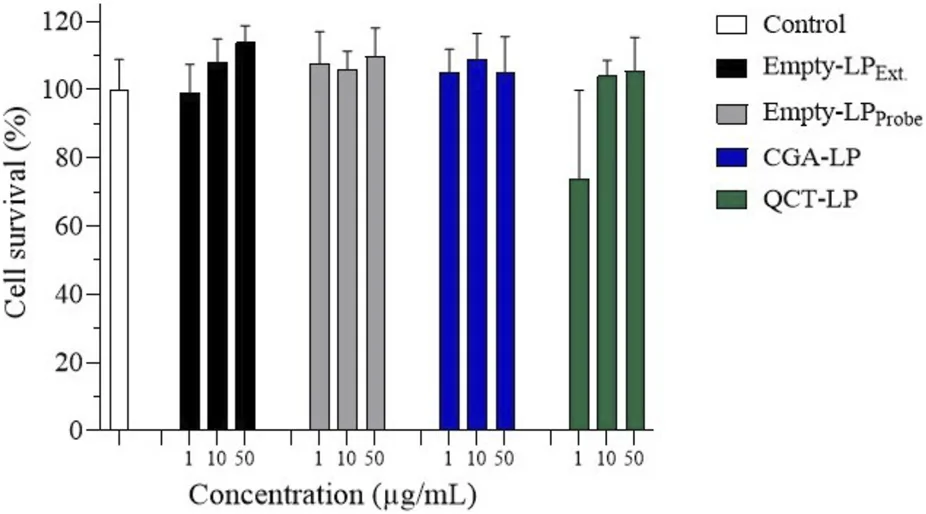
Evaluation of cytotoxicity of liposomal formulations in murine macrophages. Cell viability is expressed relative to untreated control cells (100%), which were maintained in complete medium. Data are presented as mean ± SD displayed as error bars (*n* = 3).

Biocompatibility is important to consider in the development of drug delivery systems (Upare et al., 2025), particularly for wound healing applications where macrophages play an important role (Gandolfi et al., 2024). The potential cytotoxicity of Empty-LP_Ext._, Empty-LP_Probe_, CGA-LP, and QCT-LP was assessed on murine macrophages, and the results are presented in Figure 3. Almost all formulations maintained the cell viability above 80%, which is generally regarded as a non-toxic threshold (International Organization for Standardization–ISO, 2009; Guillot et al., 2023). The Empty-LP_Ext._ and CGA-LP were seemingly safe, consistent with previous findings showing non-toxic or even proliferative effects of CGA on macrophages and keratinocytes across a range of concentrations (Kim et al., 2017; Huang H. et al., 2023). Similarly, Empty-LP_Probe_ and QCT-LP showed no adverse effects on macrophage viability at lipid concentrations up to 50 μg/mL, corresponding to QCT concentrations of 2.45 μg/mL. A minor reduction in viability was observed at the lowest QCT-LP concentration. However, these findings align with prior studies reporting high viability in various cell lines for QCT-LPs and related flavonoid liposomal formulations (Giordani et al., 2020; Liu et al., 2024). In contrast, free QCT at equivalent concentrations exhibited cytotoxic effects, as shown in Supplementary Figure S1.

These results support the biocompatibility of both CGA-LPs and QCT-LPs, reinforcing that anionic and neutral liposomes with compositions similar to cell membranes generally exhibit low cytotoxicity (Tseu and Kamaruzaman, 2023).

### 3.5 Assessment of anti-inflammatory potential

**FIGURE 4:**
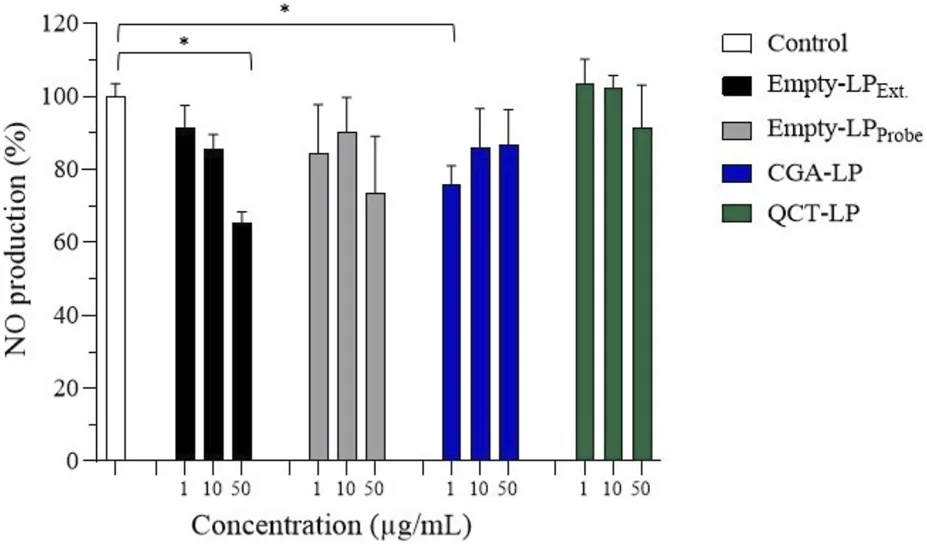
Evaluation of anti-inflammatory activity of liposomal formulations in murine macrophages. Nitric oxide (NO) production was measured in LPS-stimulated RAW 264.7 cells treated with liposomal formulations at lipid concentrations of 1, 10, and 50 μg/mL. The NO levels are expressed relative to the LPS-treated control (100%). Data are presented as mean ± SD displayed as error bars (*n* = 3) *p ≤ 0.05.

The anti-inflammatory activity of the liposomal formulations was evaluated by measuring nitric oxide (NO) production in LPS-stimulated murine macrophage RAW 264.7 cells (Figure 4). The Empty-LP_Ext._ induced a concentration-dependent reduction in NO production, with a statistically significant decrease at 50 μg/mL compared to the LPS control, corresponding to approximately 65% of NO production at the highest concentration tested. In contrast, the Empty-LP_Probe_ showed a weaker and less consistent reduction in NO levels across the tested concentrations. However, the NO production is seemingly reducing by increase of concentrations.

Moreover, the CGA-LP also significantly reduced NO production at the lowest tested concentration (1 μg/mL) compared to the LPS control. However, increasing the concentration did not result in a further decrease in NO levels, and the overall NO production remained comparable to the lower concentrations of the corresponding empty liposomes. This suggests that while CGA-LP exerts an anti-inflammatory effect at low concentration, CGA encapsulation does not confer a pronounced additional inhibitory effect on NO production under the conditions tested. Similarly, the QCT-LP resulted in no reduction in NO production relative to the LPS control. These results indicate that the reduction in NO production observed at low concentrations is most likely primarily associated with the liposomal formulations themselves as seen in previous studies (Cauzzo et al., 2020; Hemmingsen et al., 2021). The observed response likely also depends on the formulation-specific characteristics and the experimental conditions employed.

In contrast, Giordani *et al.* reported a strong and concentration-dependent inhibition of NO production in LPS-stimulated RAW 264.7 macrophages treated with QCT- or gallic acid-loaded liposomes, with significantly greater effects than those observed for plain liposomes (Giordani et al., 2020). Hong *et al.* reported a strong, concentration-dependent inhibition of NO production in LPS-stimulated RAW 264.7 macrophages using *I. britannica* extract-loaded liposomes (Hong et al., 2023). Differences in formulation composition and experimental design could contribute to the variation in outcomes observed across studies.

### 3.6 Antimicrobial evaluation

#### 3.6.1 Broth microdilution assay

The antibacterial activity of liposomal formulations assessed against *S. aureus* MSSA 476 are summarized in Table 3. The MIC for CGA was not within the tested range for either preparation. A slight inhibition of 25.5% was determined for the highest concentration of free CGA, 240 μg/mL, based on OD measurements. Reports on MIC for CGA against *S. aureus* varies widely, ranging from 40 μg/mL to 4,000 μg/mL depending on the strain and method (Lou et al., 2011; Su et al., 2014; Adamczak et al., 2020), supporting that the MIC is outside the testing range used in this study.

**TABLE 3:** Minimum inhibitory concentrations (MIC) of all liposome formulations against *Staphylococcus aureus*.

Sample/formulation	MIC (µg/mL)
Empty-LP_Ext_	N/A
Empty-LP_Probe_	N/A
CGA	N/A
CGA-LP	N/A
QCT	N/A^¥^
QCT-LP	61.25

N/A: data not attainable in test range.

^¥^: Due to limited solubility, 10 μg/mL was the highest non-precipitating concentration (using <1% DMSO) and therefore the maximum concentration tested.

Due to limited solubility, free QCT could only be tested up to 10 μg/mL in this assay, the highest non-precipitating concentration, at which it achieved 25.5% inhibition. For the liposomal preparation of QCT, MIC was determined to 61.25 μg/mL. Compared to previous studies, QCT in different nanocarriers has shown variable MICs. Al-Samydai *et al.* reported a MIC of 2.9 μg/mL for QCT-LPs against *S. aureus* ATCC 43300 (Al-Samydai et al., 2023), whereas free QCT exhibited MIC values of 16-≥1,024 μg/mL depending on *S. aureus* strain (dos Santos et al., 2021; Morimoto et al., 2023).

Variations in reported MICs of the polyphenols CGA and QCT likely reflect variations in nanocarrier design and bacterial strain. Further evaluation against a larger panel of bacterial strains could clarify the potential of these liposomal formulations for pharmaceutical antimicrobial applications.

#### 3.6.2 Isothermal microcalorimetry

Since no antibacterial effect was observed for GCA-LP in the broth microdilution assay, the CGA formulations were further examined to determine whether changes in bacterial growth dynamics in response to CGA could be detected by IMC.

**FIGURE 5:**
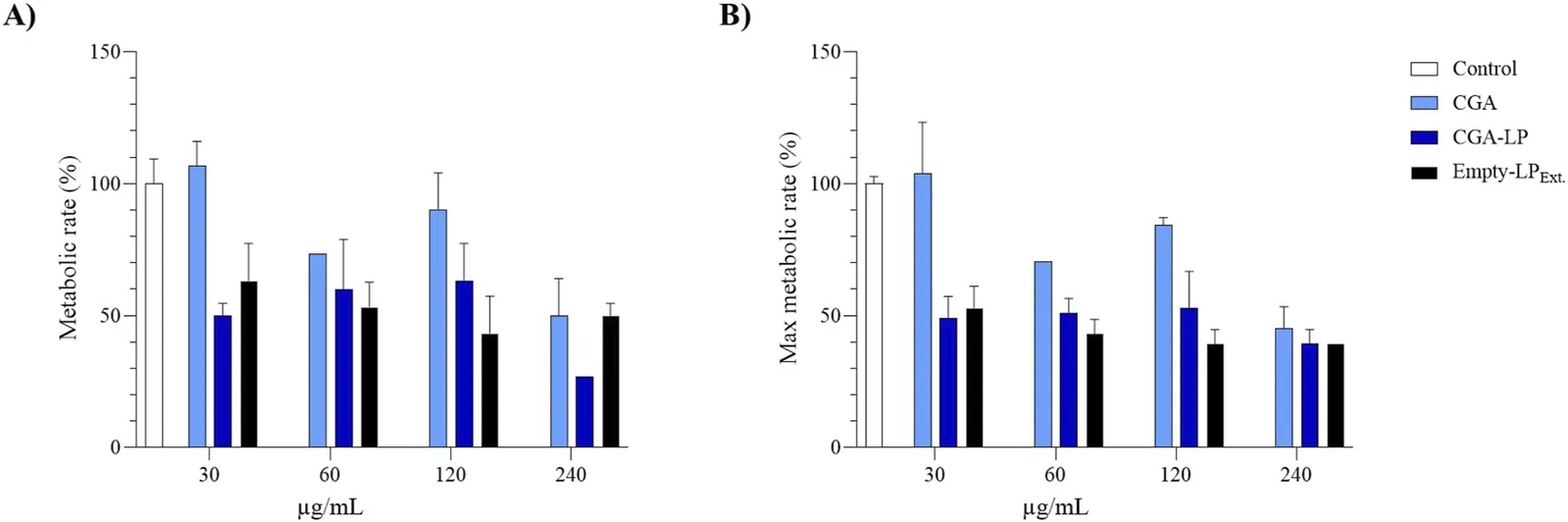
Effects of Empty-LP_Ext._, free CGA, and CGA-LP on the metabolic activity of *Staphylococcus aureus*. **(A)** Total metabolic rate and **(B)** maximum metabolic rate at CGA concentrations of 30, 60, 120, and 240 μg/mL. Metabolic activity is expressed as a percentage of the positive control (100%). Results are presented as mean ± SD (*n* = 2).

Conventional antibacterial testing methods, such as broth microdilution assays, are time-consuming, susceptible to variability, and only shows end-point results. Isothermal microcalorimeters, such as the CalScreener™, could serve as a novel alternative by enabling real-time monitoring of bacterial metabolic activity, which acts as a proxy for growth rate (Bayode et al., 2022). In our study, *S. aureus* MSSA 476 treated with CGA-LP, Empty-LP_Ext._, or free CGA showed reductions in both total metabolic rate and maximum metabolic rate compared to untreated controls (Figures 5A, B). Notably, CGA-LP induced a greater reduction in total metabolic rate than free CGA, while both CGA-LP and Empty-LP_Ext._ decreased maximum metabolic activity by ∼50% across all tested concentrations.

While free CGA relatively consistently reduced metabolic activity, CGA-LP showed a more pronounced suppression of metabolic rate at lower concentrations (30–120 μg/mL), suggesting that entrapment might enhance the interaction or uptake of CGA by bacterial cells at sub-inhibitory concentrations. Interestingly, Empty-LP_Ext._ alone also decreased measurable metabolic activity, particularly at lower concentrations, which could imply that components of the liposomal carrier exert subtle metabolic pressure or changes on *S. aureus* independently of the active compound. The observation that both CGA-LP and Empty-LP_Ext._ produced similar reductions in maximum metabolic rate at several concentrations could suggest that the delivery system might affect peak bacterial metabolic performance rather than cumulative growth. This could be a result of internalization of lipids from the liposomes, as described by Scheeder *et al.* for *Bacillus subtilis* (Scheeder et al., 2023), causing intracellular interactions that affect the metabolic activity. These results point toward the added value of IMC in revealing nuanced metabolic effects that are not captured by endpoint MIC measurements, suggesting potential impacts of formulations that could influence bacterial physiology and response to treatments.

These findings are in contrast with the results of the broth microdilution assay, where free CGA appeared more effective. This discrepancy likely reflects the different readouts of the two methods, namely, metabolic activity could persist in bacteria even when growth is inhibited, and conversely, decreases in metabolic activity do not always correspond directly to reductions in viable bacteria. Nevertheless, the CalScreener™ data suggest that CGA-LP has the potential to alter bacterial metabolism and might provide complementary insights to conventional MIC measurements (Morazzoni et al., 2024).

Sousa *et al.* investigated polymyxin B-stabilized micelles against bacterial biofilms. Using IMC with the CalScreener™, the authors demonstrated a clear dose-dependent delay in metabolic activity and a reduction in growth kinetics within biofilms, confirming the bactericidal activity of polymyxin B. This study highlights the importance of considering bacterial biofilms, known for their higher tolerance to antimicrobial compounds (Sousa et al., 2023). In planktonic bacteria, Garmendia Urdalleta *et al.* investigated the antibacterial activity of titanium implants fabricated by additive manufacturing and subsequently functionalized via plasma electrolytic oxidation in the presence of silver nanoparticles against *S. aureus* and *E. coli* using the CalScreener™. In this study, planktonic bacterial metabolic activity was monitored in real time, revealing minimal effects on *S. aureus* but a delayed and reduced metabolic response for *Escherichia coli* on the silver-containing surfaces. However, SEM imaging confirmed that silver nanoparticle incorporation limited surface colonization, demonstrating that these implants could inhibit bacterial growth (Garmendia Urdalleta et al., 2023). While few studies to date have applied this technique, especially including delivery systems or advanced pharmaceutical systems, findings are indicative of the value of real-time metabolic monitoring in characterizing antimicrobial activity which could be beneficial in development of pharmaceutical innovations.

## 4 Conclusion

Liposomal entrapment of CGA and QCT was successful and enabled sustained release from the delivery system, in which CGA-LPs exhibited a lower cumulative release, whereas QCT-LPs showed a higher cumulative release. Additionally, QCT-LP demonstrated measurable antibacterial activity against *S. aureus* in a conventional broth microdilution assay, whereas CGA-LP altered the metabolic activity of *S. aureus* via IMC. Both formulations demonstrated suitable biocompatibility with murine macrophages, supporting their suitability in topical use. These findings indicate that liposomal encapsulation could modulate the delivery and biological performance of polyphenols and support their further development as topical delivery systems in chronic wound management. Importantly, while the present study provides evidence of encouraging release characteristics, antimicrobial activity, and biocompatibility, direct evaluation of wound-healing efficacy in biologically relevant wound models remains necessary to confirm the therapeutic relevance of these formulations. However, additional studies in relevant *in vitro* and *in vivo* wound models will be required to establish their effects on oxidative stress, inflammation, and wound healing processes.

## Data Availability

The raw data supporting the conclusions of this article will be made available by the authors, without undue reservation.

## References

[B1] Adamczak A. Ożarowski M. Karpiński T. M. (2020). Antibacterial activity of some flavonoids and organic acids widely distributed in plants. J. Clin. Med. 9 (1), 109. 10.3390/jcm9010109 31906141 PMC7019947

[B2] Akombaetwa N. Ilangala A. B. Thom L. Memvanga P. B. Witika B. A. Buya A. B. (2023). Current advances in lipid nanosystems intended for topical and transdermal drug delivery applications. Pharmaceutics 15 (2), 656. 10.3390/pharmaceutics15020656 36839978 PMC9967415

[B3] Al-Samydai A. Al Qaraleh M. Al Azzam K. M. Mayyas A. Nsairat H. Abu Hajleh M. N. (2023). Formulating co-loaded nanoliposomes with gallic acid and quercetin for enhanced cancer therapy. Heliyon 9 (6), e17267. 10.1016/j.heliyon.2023.e17267 37408902 PMC10319229

[B4] Aytekin A. A. Tuncay Tanrıverdi S. Aydın Köse F. Kart D. Eroğlu İ. Özer Ö. (2020). Propolis loaded liposomes: evaluation of antimicrobial and antioxidant activities. J. Liposome Res. 30 (2), 107–116. 10.1080/08982104.2019.1599012 30913939

[B5] Balouiri M. Sadiki M. Ibnsouda S. K. (2016). Methods for *in vitro* evaluating antimicrobial activity: a review. J. Pharm. Anal. 6 (2), 71–79. 10.1016/j.jpha.2015.11.005 29403965 PMC5762448

[B6] Bayode M. T. Alabi M. A. Babatunde O. J. Sadibo M. E. Lawani B. T. Okiti A. F. (2022). Isothermal microcalorimetry (IMC) calscreener: automated peculiarities of antimicrobial therapy and metabolism depth of multidrug resistant bacteria. Bull. Natl. Res. Cent. 46 (1), 149. 10.1186/s42269-022-00841-w

[B7] Braido B. Rukavina Z. Grimstad Ø. Franzè S. Cilurzo F. Vanić Ž. (2025). Liposomes-in-hydrogel for topical drug delivery: mechanical, kinetic, and biological insights. J. Drug Deliv. Sci. Technol. 113, 107380. 10.1016/j.jddst.2025.107380

[B8] Cauzzo J. Nystad M. Holsæter A. M. Basnet P. Škalko-Basnet N. (2020). Following the fate of dye-containing liposomes *in vitro* . Int. J. Mol. Sci. 21 (14), 4847. 10.3390/ijms21144847 32659908 PMC7402323

[B9] Chen G. Li D. Jin Y. Zhang W. Teng L. Bunt C. (2014). Deformable liposomes by reverse-phase evaporation method for an enhanced skin delivery of (+)-catechin. Drug Dev. Ind. Pharm. 40 (2), 260–265. 10.3109/03639045.2012.756512 23356860

[B10] Choi Y.-E. Choi S.-I. Han X. Men X. Jang G.-W. Kwon H.-Y. (2020). Radical scavenging-linked anti-adipogenic activity of Aster scaber ethanolic extract and its bioactive compound. Antioxidants 9 (12), 1290. 10.3390/antiox9121290 33339396 PMC7766398

[B11] Clinical Laboratory Standards Institute (2012). Methods for Dilution Antimicrobial Susceptibility Tests for Bacteria that Grow Aerobically - Ninth Edition: Approved Standard M07-A9.

[B12] Comino-Sanz I. M. López-Franco M. D. Castro B. Pancorbo-Hidalgo P. L. (2021). The role of antioxidants on wound healing: a review of the current evidence. J. Clin. Med. 10 (16), 3558. 10.3390/jcm10163558 34441854 PMC8397081

[B13] Dandamudi M. Mcloughlin P. Behl G. Coffey L. Chauhan A. Kent D. (2024). Investigation of novel combination therapy for age-related macular degeneration on ARPE-19 cells. Front. Drug Deliv. 4 (4 - 2024), 1337686. 10.3389/fddev.2024.1337686 40836983 PMC12363285

[B14] Dos Santos J. F. S. Tintino S. R. Da Silva A. R. P. Dos S. Barbosa C. R. Scherf J. R. (2021). Enhancement of the antibiotic activity by quercetin against *Staphylococcus aureus* efflux pumps. J. Bioenerg. Biomembr. 53 (2), 157–167. 10.1007/s10863-021-09886-4 33683535

[B15] Dragicevic N. Maibach H. I. (2024). Liposomes and other nanocarriers for the treatment of acne vulgaris: improved therapeutic efficacy and skin tolerability. Pharmaceutics 16 (3), 309. 10.3390/pharmaceutics16030309 38543203 PMC10974767

[B16] Eloy J. O. Claro De Souza M. Petrilli R. Barcellos J. P. A. Lee R. J. Marchetti J. M. (2014). Liposomes as carriers of hydrophilic small molecule drugs: strategies to enhance encapsulation and delivery. Colloids Surf. B 123, 345–363. 10.1016/j.colsurfb.2014.09.029 25280609

[B17] Falanga V. Isseroff R. R. Soulika A. M. Romanelli M. Margolis D. Kapp S. (2022). Chronic wounds. Nat. Rev. Dis. Prim. 8 (1), 50. 10.1038/s41572-022-00377-3 35864102 PMC10352385

[B18] Falcone M. De Angelis B. Pea F. Scalise A. Stefani S. Tasinato R. (2021). Challenges in the management of chronic wound infections. J. Glob. Antimicrob. Resist. 26, 140–147. 10.1016/j.jgar.2021.05.010 34144200

[B19] Ferreira M. Ogren M. Dias J. N. R. Silva M. Gil S. Tavares L. (2021). Liposomes as antibiotic delivery systems: a promising nanotechnological strategy against antimicrobial resistance. Molecules 26 (7), 2047. 10.3390/molecules26072047 33918529 PMC8038399

[B20] Gandolfi S. Sanouj A. Chaput B. Coste A. Sallerin B. Varin A. (2024). The role of adipose tissue-derived stromal cells, macrophages and bioscaffolds in cutaneous wound repair. Biol. Direct. 19 (1), 85. 10.1186/s13062-024-00534-6 39343924 PMC11439310

[B21] Garmendia Urdalleta A. Van Poll M. Fahy N. Witte-Bouma J. Van Wamel W. Apachitei I. (2023). The response of human macrophages to 3D printed titanium antibacterial implants does not affect the osteogenic differentiation of hMSCs. Front. Bioeng. Biotechnol. 11 - 2023, 1176534. 10.3389/fbioe.2023.1176534 37415788 PMC10319998

[B22] Giordani B. Basnet P. Mishchenko E. Luppi B. Škalko-Basnet N. (2020). Utilizing liposomal quercetin and gallic acid in localized treatment of vaginal candida infections. Pharmaceutics 12 (1), 9. 10.3390/pharmaceutics12010009 31861805 PMC7023398

[B23] Guillot A. J. Martínez-Navarrete M. Garrigues T. M. Melero A. (2023). Skin drug delivery using lipid vesicles: a starting guideline for their development. J. Control. Release 355, 624–654. 10.1016/j.jconrel.2023.02.006 36775245

[B24] Hemmingsen L. M. Julin K. Ahsan L. Basnet P. Johannessen M. Škalko-Basnet N. (2021). Chitosomes-in-chitosan hydrogel for acute skin injuries: prevention and infection control. Mar. Drugs 19 (5), 269. 10.3390/md19050269 34065943 PMC8150996

[B25] Hemmingsen L. M. Panchai P. Julin K. Basnet P. Nystad M. Johannessen M. (2022). Chitosan-based delivery system enhances antimicrobial activity of chlorhexidine. Front. Microbiol. 13, 1023083. 10.3389/fmicb.2022.1023083 36246245 PMC9557914

[B26] Hong C. R. Lee E. H. Jung Y. H. Lee J.-H. Paik H.-D. Hong S.-C. (2023). Development and characterization of Inula britannica extract-loaded liposomes: potential as anti-inflammatory functional food ingredients. Antioxidants 12 (8), 1636. 10.3390/antiox12081636 37627631 PMC10451523

[B27] Hu S. Zhao R. Chi X. Chen T. Li Y. Xu Y. (2024). Unleashing the power of chlorogenic acid: exploring its potential in nutrition delivery and the food industry. Food Funct. 15 (9), 4741–4762. 10.1039/D4FO00059E 38629635

[B28] Huang H. Chen L. Hou Y. He W. Wang X. Zhang D. (2023a). Self-assembly of chlorogenic acid into hydrogel for accelerating wound healing. Colloids Surf. B 228, 113440. 10.1016/j.colsurfb.2023.113440 37421764

[B29] Huang J. Xie M. He L. Song X. Cao T. (2023b). Chlorogenic acid: a review on its mechanisms of anti-inflammation, disease treatment, and related delivery systems. Front. Pharmacol. 14, 1218015. 10.3389/fphar.2023.1218015 37781708 PMC10534970

[B30] Hupfeld S. Holsæter A. M. Skar M. Frantzen C. B. Brandl M. (2006). Liposome size analysis by dynamic/static light scattering upon size exclusion-/field flow-fractionation. J. Nanosci. Nanotechnol. 6 (9-10), 3025–3031. 10.1166/jnn.2006.454 17048514

[B31] Hurlow J. Bowler P. G. (2022). Acute and chronic wound infections: microbiological, immunological, clinical and therapeutic distinctions. J. Wound Care 31 (5), 436–445. 10.12968/jowc.2022.31.5.436 35579319

[B32] International Organization for Standardization – ISO (2009). “ISO, 10993-5:2009 biological evaluation of medical devices,” in Part 5: Tests for *in vitro* Cytotoxicity (Geneva, Switzerland: International Organization for Standardization - ISO).

[B33] Izu G. O. Mfotie Njoya E. Tabakam G. T. Nambooze J. Otukile K. P. Tsoeu S. E. (2024). Unravelling the influence of chlorogenic acid on the antioxidant phytochemistry of avocado (Persea americana mill.) fruit peel. Antioxidants 13 (4), 456. 10.3390/antiox13040456 38671904 PMC11047442

[B34] Jøraholmen M. W. Basnet P. Tostrup M. J. Moueffaq S. Škalko-Basnet N. (2019). Localized therapy of vaginal infections and inflammation: liposomes-in-hydrogel delivery system for polyphenols. Pharmaceutics 11 (2), 53. 10.3390/pharmaceutics11020053 30691199 PMC6410284

[B35] Kamini Puri D. (2026). QbD assisted formulation, optimization and characterization of psoralen phytosome prepared through thin film hydration method. Drug Dev. Ind. Pharm. 52 (2), 291–312. 10.1080/03639045.2025.2593532 41283982

[B36] Kapoor D. U. Khanday M. A. Pareek A. Prajapati B. G. (2025). Reengineering infection treatment: liposome-based innovations in antibacterial delivery. Results Chem. 16, 102417. 10.1016/j.rechem.2025.102417

[B37] Karonen M. (2022). Insights into polyphenol–lipid interactions: chemical methods, molecular aspects and their effects on membrane structures. Plants 11 (14), 1809. 10.3390/plants11141809 35890443 PMC9317924

[B38] Kim S. H. Park S. Y. Park Y. L. Myung D. S. Rew J. S. Joo Y. E. (2017). Chlorogenic acid suppresses lipopolysaccharide-induced nitric oxide and interleukin-1β expression by inhibiting JAK2/STAT3 activation in RAW264.7 cells. Mol. Med. Rep. 16 (6), 9224–9232. 10.3892/mmr.2017.7686 28990048

[B39] Kolluri R. Lugli M. Villalba L. Varcoe R. Maleti O. Gallardo F. (2022). An estimate of the economic burden of venous leg ulcers associated with deep venous disease. Vasc. Med. 27 (1), 63–72. 10.1177/1358863X211028298 34392750 PMC8808361

[B40] Li C. Zhang X. Huang X. Wang X. Liao G. Chen Z. (2013). Preparation and characterization of flexible nanoliposomes loaded with daptomycin, a novel antibiotic, for topical skin therapy. Int. J. Nanomed. 8, 1285–1292. 10.2147/IJN.S41695 23569376 PMC3615926

[B41] Li X. Zhu S. Yin P. Zhang S. Xu J. Zhang Q. (2021). Combination immunotherapy of chlorogenic acid liposomes modified with sialic acid and PD-1 blockers effectively enhances the anti-tumor immune response and therapeutic effects. Drug Deliv. 28 (1), 1849–1860. 10.1080/10717544.2021.1971797 34515617 PMC8439241

[B42] Li Q.-Q. Yan J.-H. Zhou Z.-E. Geng X. Xiong J.-H. (2024). Enhanced anti-inflammatory activity of chlorogenic acid via folic acid-TPGS-modified liposomes encapsulation: characterization and *in vivo* evaluation on colitis mice. Front. Pharmacol. 15, 15–2024. 10.3389/fphar.2024.1437773 39246657 PMC11377334

[B43] Li R. Liu B. Wang C. Sun Z. Lv S. Tian L. (2025). A pH-responsive charge-reversal liposome with enhanced biofilm penetration for multimodal synergistic therapy of bacterial infections. BMEMat 4, e70014. 10.1002/bmm2.70014

[B44] Liu X. Yu S. Lu X. Zhang Y. Zhong H. Zhou Z. (2024). Optimization of preparation conditions for quercetin nanoliposomes using response surface methodology and evaluation of their stability. ACS Omega 9 (15), 17154–17162. 10.1021/acsomega.3c09892 38645336 PMC11024936

[B45] Lou Z. Wang H. Zhu S. Ma C. Wang Z. (2011). Antibacterial activity and mechanism of action of chlorogenic acid. J. Food Sci. 76 (6), M398–M403. 10.1111/j.1750-3841.2011.02213.x 22417510

[B46] Luo M. Oomah B. D. Akoetey W. Zhang Y. Daneshfozoun H. Hosseinian F. (2025). Liposomes as sustainable delivery systems in food, cosmetic, and pharmaceutical applications. J. Am. Oil Chem. Soc. 102 (3), 547–568. 10.1002/aocs.12907

[B47] Mardhiah Adib Z. Ghanbarzadeh S. Kouhsoltani M. Yari Khosroshahi A. Hamishehkar H. (2016). The effect of particle size on the deposition of solid lipid nanoparticles in different skin layers: a histological study. Adv. Pharm. Bull. 6 (1), 31–36. 10.15171/apb.2016.06 27123415 PMC4845546

[B48] Meers P. Neville M. Malinin V. Scotto A. W. Sardaryan G. Kurumunda R. (2008). Biofilm penetration, triggered release and *in vivo* activity of inhaled liposomal amikacin in chronic *Pseudomonas aeruginosa* lung infections. J. Antimicrob. Chemother. 61 (4), 859–868. 10.1093/jac/dkn059 18305202

[B49] Morazzoni C. Sirel M. Allesina S. Veses Garcia M. Kragh K. Pane M. (2024). Proof of concept: real-time viability and metabolic profiling of probiotics with isothermal microcalorimetry. Front. Microbiol. 15, 1391688. 10.3389/fmicb.2024.1391688 38962141 PMC11220157

[B50] Morimoto Y. Aiba Y. Miyanaga K. Hishinuma T. Cui L. Baba T. (2023). CID12261165, a flavonoid compound as antibacterial agents against quinolone-resistant *Staphylococcus aureus* . Sci. Rep. 13 (1), 1725. 10.1038/s41598-023-28859-8 36720958 PMC9889749

[B51] Nazari M. Shokoohizadeh L. Taheri M. (2025). Natural products in the treatment of diabetic foot infection. Eur. J. Med. Res. 30 (1), 8. 10.1186/s40001-024-02255-y 39773682 PMC11705749

[B52] Nsairat H. Khater D. Sayed U. Odeh F. Al Bawab A. Alshaer W. (2022). Liposomes: structure, composition, types, and clinical applications. Heliyon 8 (5), e09394. 10.1016/j.heliyon.2022.e09394 35600452 PMC9118483

[B53] Ohnishi M. Morishita H. Iwahashi H. Toda S. Shirataki Y. Kimura M. (1994). Inhibitory effects of chlorogenic acids on linoleic acid peroxidation and haemolysis. Phytochemistry 36 (3), 579–583. 10.1016/S0031-9422(00)89778-2

[B54] Raina N. Rani R. Thakur V. K. Gupta M. (2023). New insights in topical drug delivery for skin disorders: from a nanotechnological perspective. ACS Omega 8 (22), 19145–19167. 10.1021/acsomega.2c08016 37305231 PMC10249123

[B55] Scheeder A. Brockhoff M. Ward E. N. Kaminski Schierle G. S. Mela I. Kaminski C. F. (2023). Molecular mechanisms of cationic fusogenic liposome interactions with bacterial envelopes. J. Am. Chem. Soc. 145 (51), 28240–28250. 10.1021/jacs.3c11463 38085801 PMC10755748

[B56] Sen C. K. (2023). Human wound and its burden: updated 2022 compendium of estimates. Adv. Wound Care 12 (12), 657–670. 10.1089/wound.2023.0150 37756368 PMC10615092

[B57] Shabir I. Kumar Pandey V. Shams R. Dar A. H. Dash K. K. Khan S. A. (2022). Promising bioactive properties of quercetin for potential food applications and health benefits: a review. Front. Nutr. 9, 9–2022. 10.3389/fnut.2022.999752 36532555 PMC9748429

[B58] Sharma A. Shankar R. Yadav A. K. Pratap A. Ansari M. A. Srivastava V. (2024). Burden of chronic nonhealing wounds: an overview of the worldwide humanistic and economic burden to the healthcare system. Int. J. Low. Extrem. Wounds 25, 15347346241246339. 10.1177/15347346241246339 38659348

[B59] Smith M. C. Crist R. M. Clogston J. D. Mcneil S. E. (2017). Zeta potential: a case study of cationic, anionic, and neutral liposomes. Anal. Bioanal. Chem. 409 (24), 5779–5787. 10.1007/s00216-017-0527-z 28762066

[B60] Sousa A. Borøy V. Bæverud A. Julin K. Bayer A. Strøm M. (2023). Polymyxin B stabilized DNA micelles for sustained antibacterial and antibiofilm activity against *P. aeruginosa* . J. Mater. Chem. B 11, 7972–7985. 10.1039/D3TB00704A 37505112

[B61] Su Y. Ma L. Wen Y. Wang H. Zhang S. (2014). Studies of the *in vitro* antibacterial activities of several polyphenols against clinical isolates of methicillin-resistant *Staphylococcus aureus* . Molecules 19 (8), 12630–12639. 10.3390/molecules190812630 25153875 PMC6271679

[B62] Tang K. W. K. Millar B. C. Moore J. E. (2023). Antimicrobial resistance (AMR). Br. J. Biomed. Sci. 80 - 2023, 11387. 10.3389/bjbs.2023.11387 37448857 PMC10336207

[B63] Tellapragada C. Hasan B. Antonelli A. Maruri A. De Vogel C. Gijón D. (2020). Isothermal microcalorimetry minimal inhibitory concentration testing in extensively drug resistant Gram-negative bacilli: a multicentre study. Clin. Microbiol. Infect. 26 (10), 1413.e1411–1413.e1417. 10.1016/j.cmi.2020.01.026 32006694

[B64] Thabet Y. Elsabahy M. Eissa N. G. (2022). Methods for preparation of niosomes: a focus on thin-film hydration method. Methods 199, 9–15. 10.1016/j.ymeth.2021.05.004 34000392

[B65] Tomazoni E. I. Nihei O. K. (2025). Clinical, epidemiological, nutritional and mental health characteristics of patients with chronic wounds. Rev. Nutr. 38, e240131. 10.1590/1678-9865202538e240131

[B66] Trivedi H. R. Puranik P. K. (2023). Chlorogenic acid loaded niosomes and proniosomes: *in vitro* antioxidant and antibacterial activities with efficacy in wound healing. Digit. Chin. Med. 6 (2), 170–188. 10.1016/j.dcmed.2023.07.007

[B67] Tseu G. Y. W. Kamaruzaman K. A. (2023). A review of different types of liposomes and their advancements as a form of gene therapy treatment for breast cancer. Molecules 28 (3), 1498. 10.3390/molecules28031498 36771161 PMC9920768

[B68] Uberoi A. Mccready-Vangi A. Grice E. A. (2024). The wound microbiota: microbial mechanisms of impaired wound healing and infection. Nat. Rev. Microbiol. 22 (8), 507–521. 10.1038/s41579-024-01035-z 38575708

[B69] Upare M. M. Thorawade K. M. Mishra A. P. Nigam M. Waranuch N. (2025). Lipid-based nanocarriers in topical applications for skin infections. Curr. Opin. Pharmacol. 83, 102541. 10.1016/j.coph.2025.102541 40466274

[B70] Wang W. Lu K.-J. Yu C.-H. Huang Q.-L. Du Y.-Z. (2019). Nano-drug delivery systems in wound treatment and skin regeneration. J. Nanobiotechnology 17 (1), 82. 10.1186/s12951-019-0514-y 31291960 PMC6617859

[B71] Wang S. Li Y. Meng X. Chen S. Huang D. Xia Y. (2021a). Antioxidant activities of chlorogenic acid derivatives with different acyl donor chain lengths and their stabilities during *in vitro* simulated gastrointestinal digestion. Food Chem. 357, 129904. 10.1016/j.foodchem.2021.129904 33915469

[B72] Wang Z. Liu L. Yin W. Liu Z. Shi L. Tang M. (2021b). A novel drug delivery system: the encapsulation of naringenin in metal-organic frameworks into liposomes. AAPS PharmSciTech 22 (2), 61. 10.1208/s12249-021-01927-w 33527250

[B73] Wang R. Peng J. Shi X. Cao S. Xu Y. Xiao G. (2022). Change in membrane fluidity induced by polyphenols is highly dependent on the position and number of galloyl groups. Biochim. Biophys. Acta - Biomembr. 1864 (11), 184015. 10.1016/j.bbamem.2022.184015 35914569

[B74] Wolcott R. D. Hanson J. D. Rees E. J. Koenig L. D. Phillips C. D. Wolcott R. A. (2016). Analysis of the chronic wound microbiota of 2,963 patients by 16S rDNA pyrosequencing. Wound Repair. Regen. 24 (1), 163–174. 10.1111/wrr.12370 26463872

[B75] Xiang Y. Xiang M. Mao Y. Huang L. He Q. Dong Y. (2025). Insights into structure-antioxidant activity relationships of polyphenol-phospholipid complexes: the effect of hydrogen bonds formed by phenolic hydroxyl groups. Food Chem. 485, 144471. 10.1016/j.foodchem.2025.144471 40306058

