# Supplementary material for: Harnessing liposomal delivery to boost polyphenols’ potential in chronic wound therapy
Source: Front Drug Deliv. 2026 Aug 21;6:1839747. doi: 10.3389/fddev.2026.1839747 (PMC13541760; doi:10.3389/fddev.2026.1839747)
Supplement: Supplementary file 1 [file Supplementaryfile1.docx]

Supplementary Material

# 1 Supplementary Results

## 1.1 Cell toxicity of free quercetin

Figure S1 shows that free QCT in DMSO is highly cytotoxic to macrophages, with cell survival dropping below 5% even at the lowest concentration tested (0.049 µg/mL QCT). In contrast, the control and DMSO-only (due to the poor water solubility of QCT) treatments maintain high cell viability, indicating that the solvent itself is not harmful. Importantly, QCT when loaded in liposomes did not reduce macrophage viability, suggesting that liposomal encapsulation effectively prevents QCT-induced cytotoxicity and makes it biocompatible for these cells.

**Figure S1.** Evaluation of cytotoxicity of quercetin (QCT) in murine macrophages. Cells were treated with QCT dissolved in DMSO at concentrations corresponding to lipid concentrations of 1, 10, and 50 µg/mL or 0.049, 0.49, and 2.45 µg/mL QCT. Controls included untreated cells and cells treated with DMSO alone. Cell viability is expressed relative to untreated control cells (100%). Data are presented as mean ± SD (*n = 3*).
